# NIR Imaging of the Integrin-Rich Head and Neck Squamous Cell Carcinoma Using Ternary Copper Indium Selenide/Zinc Sulfide-Based Quantum Dots

**DOI:** 10.3390/cancers12123727

**Published:** 2020-12-11

**Authors:** Ilya Yakavets, Aurelie Francois, Maelle Guiot, Nicolas Lequeux, Alexandra Fragola, Thomas Pons, Lina Bezdetnaya, Frédéric Marchal

**Affiliations:** 1 Research Department, Institut de Cancérologie de Lorraine, Université de Lorraine, CNRS UMR7039 CRAN, 6 avenue de Bourgogne, 54519 Vandœuvre-lès-Nancy, France; i.yakavets@nancy.unicancer.fr (I.Y.); a.francois@nancy.unicancer.fr (A.F.); maelle.guiot@outlook.fr (M.G.); f.marchal@nancy.unicancer.fr (F.M.); 2 Laboratoire de Physique et d’Étude des matériaux (LPEM, UMR 8213), ESPCI Paris, PSL University, CNRS, Sorbonne University, 10, rue Vauquelin, 75005 Paris, France; nicolas.lequeux@espci.fr (N.L.); alexandra.fragola@espci.fr (A.F.); 3 Surgical Department, Institut de Cancérologie de Lorraine, 6 avenue de Bourgogne, 54519 Vandoeuvre-lès-Nancy, France

**Keywords:** indium-based quantum dots, near-infrared imaging, bioconjugates, integrins, multicellular tumor spheroids, time-gated fluorescence microscopy

## Abstract

**Simple Summary:**

Despite improved patient outcomes in a range of cancers, the prognostic of head and neck cancers remain poor, with more than 507,000 deaths annually. The management of these cancers requires multidisciplinary treatments, including radiotherapy, chemotherapy, or targeted molecular therapy; however, surgical resection is one of the first-line treatments for patients with head and neck cancers. In order to increase the positive surgical margin rate, we proposed a tumor-specific near-infrared nanoprobe for fluorescence-guided surgery. We confirmed the high specificity of the proposed nanoprobe towards head and neck cancer cells using advanced 3D stroma-rich spheroids.

**Abstract:**

The efficient intraoperative identification of cancers requires the development of the bright, minimally-toxic, tumor-specific near-infrared (NIR) probes as contrast agents. Luminescent semiconductor quantum dots (QDs) offer several unique advantages for in vivo cellular imaging by providing bright and photostable fluorescent probes. Here, we present the synthesis of ZnCuInSe/ZnS core/shell QDs emitting in NIR (~750 nm) conjugated to NAVPNLRGDLQVLAQKVART (A20FMDV2) peptide for targeting *α_v_β_6_* integrin-rich head and neck squamous cell carcinoma (HNSCC). Integrin *α_v_β_6_* is usually not detectable in nonpathological tissues, but is highly upregulated in HNSCC. QD-A20 showed *α_v_β_6_* integrin-specific binding in two-dimension (2D) monolayer and three-dimension (3D) spheroid in vitro HNSCC models. QD-A20 exhibit limited penetration (ca. 50 µm) in stroma-rich 3D spheroids. Finally, we demonstrated the potential of these QDs by time-gated fluorescence imaging of stroma-rich 3D spheroids placed onto mm-thick tissue slices to mimic imaging conditions in tissues. Overall, QD-A20 could be considered as highly promising nanoprobes for NIR bioimaging and imaging-guided surgery.

## 1. Introduction

Worldwide, head and neck squamous cell carcinoma (HNSCC) accounts for more than 890,000 cases and more than 507,000 deaths annually [1]. The upfront surgery, as one of the first-line treatments for patients with HNSCCs along with radiotherapy or concurrent chemoradiation, requires accurate detection of tumor margins for positive postoperative prognosis. Among the important strategies for the detection of true positive margins is the evaluation of frozen sections during operation. However, this procedure is time and cost consuming and can be inaccurate [2]. In order to increase the positive surgical margins rate, the near-infrared (NIR) fluorescence-guided surgery (FGS) technique was proposed [3,4,5]. However, the efficient intraoperative identification of HNSCC requires the bright tumor-specific NIR probes as contrast agents.

Semiconductor quantum dots (QDs) represent an interesting class of fluorescent nanoparticles with exciting optical features such as a narrow tunable emission spectrum, high brightness, and photostability [6]. In particular, CuInS_2_- and CuInSe_2_-based QDs have recently emerged as a bright, less-toxic alternative to the first generation of NIR-emitting QDs based on cadmium, lead, or mercury chalcogenides [7,8,9]. Additionally, QDs possess a long fluorescence lifetime enabling efficient time-gated imaging to detect fluorescence photons from QDs and rejecting photons from tissue autofluorescence [10]. As a result, the application of time-gated imaging considerably enhances the sensitivity of the detection of QD-labeled tissues in vivo [11].

Over recent decades, integrins received major attention as biological targets for cancer therapy [12]. Integrins represent a family of surface receptors that facilitate cell–cell and cell–extracellular matrix (ECM) interactions [13]. It is acknowledged that the integrin *α_v_β_6_* plays a role in promoting a number of different pathologies, including cancer and fibrosis [14,15]. Compared to widely-used integrin *α_v_β_3_*, integrin *α_v_β_6_* is usually not detectable in nonpathological tissues, and is highly upregulated in various types of cancers, including HNSCC [16,17,18]. Given this strong specificity of *α_v_β_6_* integrin expression [16], we considered *α_v_β_6_* integrin as a highly tissue-specific target for NIR imaging of HNSCC [17,19,20].

**Figure 1:**
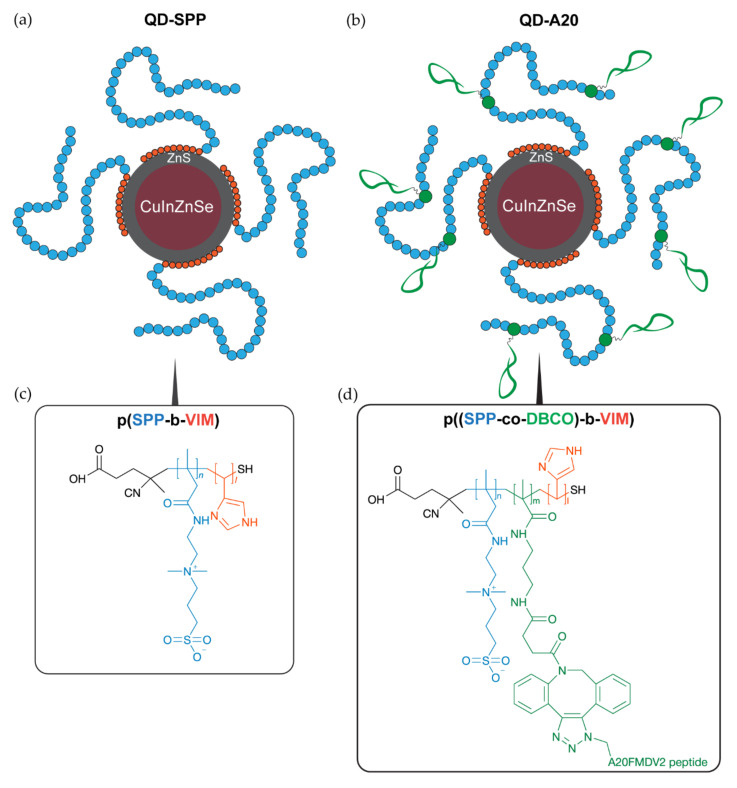
Schematics of ZnCuInSe/ZnS quantum dot (QD) structure: (**a**) Control QDs (QD-SPP) and (**b**) targeted QD-A20. (**c**,**d**) Schematics of the QD-A20 surface chemistry. Control QDs (QD-SPP) were coated with pure (**c**) p(SPP-b-VIM) block polymers, while (**d**) the p((SPP-co-APMA)-b-VIM) polymeric ligand was reacted with DBCO-NHS and with the *α_v_β_6_*-targeting peptide N_3_-A20 in QD-A20. The indicated degree of polymerization: sulfobetaine (SPP, blue, solubilization and antifouling, n ≈ 35), DBCO (green, functionalization, m ≈ 5) and imidazole (orange, anchoring, l ≈ 10).

In the present study, we reported the synthesis, characterization and targeting ability of NIR-emitting QDs coupled with *α_v_β_6_* integrin-specific peptide for noninvasive imaging of HNSCC. We have prepared NIR-emitting ZnCuInSe/ZnS core/shell QDs using a high-temperature synthesis in an organic solvent and coated with a poly(sulfobetaine-block-imidazole) block polymer (Figure 1). In previous studies, we have shown that the first imidazole block ensures a strong anchoring to the QD surface (Figure 1, in orange) thanks to its multidentate nature [21]. The second block is composed of sulfobetaine (SPP) moieties, which virtually eliminates any nonspecific interactions between the QD and proteins or other plasma components [22]. To enable conjugation with an azide terminated NAVPNLRGDLQVLAQKVART (A20FMDV2) peptide, sulfobetaine copolymer ligands were modified with dibenzocyclooctyne. A20FMDV2 peptide is able to recognize *α_v_β_6_* integrin [23,24,25]. While A20FMDV2 peptide has been used for PET imaging [23,26], its application for fluorescence imaging (i.e., conjugated with IRDye800 fluorescent dye) was reported only recently [27]. To assess the targeting ability of synthesized QDs to *α_v_β_6_* integrin-expressing cell lines, we tested QDs in stroma-rich co-culture HNSCC tumor spheroids, which recapitulate cell-ECM interactions. We tested the imaging capacity of these novel nanoprobes in tissue-like conditions using an advanced time-gated fluorescence imaging technique.

## 2. Results

### 2.1. Synthesis and Characterization of QDs

NIR-emitting ZnCuInSe/ZnS core/shell QDs coated with 100% SPP polymers were used as control samples. The functionalization of the QDs with a targeting peptide was performed by introducing a small (<15%) proportion of DBCO (in green in Figure 1), a stretched activated alkyne which reacts with N_3_ azide groups from the peptide in copper-free conditions. Here, the peptide chosen for QD targeting is derived from the A20FMDV2 (NAVPNLRGDLQVLAQKVART) sequence from the foot-and-mouth disease virus [24]. As observed in structure-function analysis studies [24], the N-terminus of this peptide is not involved in the integrin recognition, so we used it to conjugate a N_3_ group and a (GGGS)_2_ flexible linker to avoid steric hindrance issues from the SPP polymer. Colorimetric assays using N_3_-chromophores indicate that the efficiency of the conjugation of the N_3_-A20 peptide to the accessible reactive moieties on the nanocrystal surface is >90%, and that each QD is conjugated to ca. 7 peptides (Figure S1).

**Figure 2:**
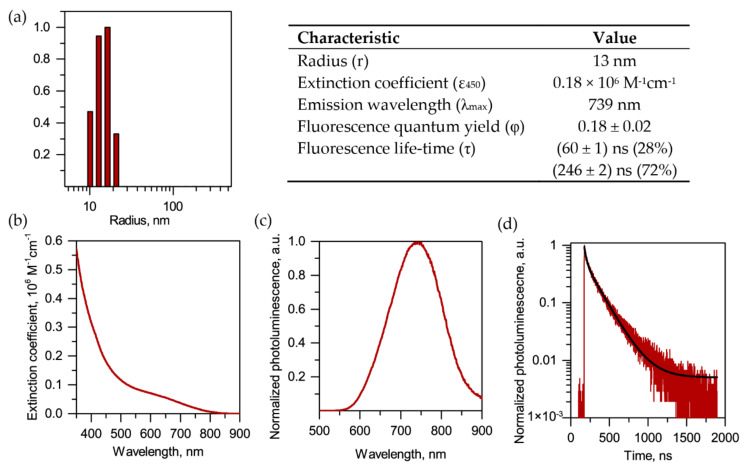
Characterization of QDs. (**a**) Dynamic light scattering (DLS) size distribution histograms and photophysical properties of QDs (right panel). (**b**) Absorbance and (**c**) fluorescence spectra of QDs. (**d**) Fluorescence emission decay curves of QDs. The black line corresponds to a biexponential fit, with the characteristic decay times and weights indicated in the right panel.

The total hydrodynamic radius, including the polymer coating and hydration layers, was 13 nm (Figure 2a), as measured by dynamic light scattering (DLS), while the semiconductor core of QDs had a diameter of ∼3–4 nm, according to the transmission electron microscopy measurements reported earlier [11,28]. During the synthesis of CuInSe cores, we incorporated zinc to increase the fluorescence quantum yield of QDs while keeping their emission wavelength in the NIR range [10,29]. QDs exhibit broad absorption and excitation spectra that extends from the UV to the NIR (ca. 350–800 nm) (Figure 2b and Figure S2). In fact, QDs intensively emit fluorescence (quantum yield = 18%) within the range of 650–800 nm (Figure 2c). Moreover, the synthesized QDs were characterized by a long component in the fluorescence decay of approximately 250 ns (Figure 2d and Figure S3), enabling the use of time-gated detection to reduce the autofluorescence background and increase imaging sensitivity [10,11]. Concerning the stability of QDs, we previously reported their excellent stability for several months in physiological buffer under the storage at 4 °C and in plasma at 37 °C [22].

### 2.2. Probing of NIR QDs as Optical Imaging Agents in 2D and 3D Models of HNSCC

#### 2.2.1. Expression of *α_v_β_6_* Integrin

**Figure 3:**
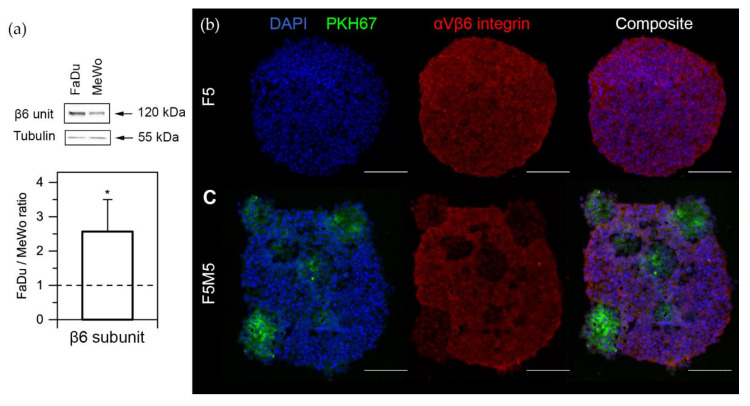
Expression of integrins in FaDu and MeWo cells. (**a**) Western blot of *β_6_* subunit expression in FaDu and MeWo cells and its quantification analysis. Data represent averages ± SD (n = 5; * *p* < 0.05, using the one-sample t-test (µ = 1)). (**b**,**c**) Typical fluorescence microscopy images of (**b**) F5 and (**c**) F5M5 spheroids cryosections, stained with antibody against *α_v_β_6_* integrins and DAPI. MeWo cells were pre-stained with PKH67 green membrane dye. Scale bar—100 μm.

The level of *α_v_β_6_* integrin expression in FaDu and MeWo monolayer cells was examined by Western blot analysis (Figure 3a). As follows from Figure 3a, the expression of *β_6_* subunit (band at 120 kDa) in 2D monolayer was much stronger in FaDu cells compared to MeWo (the ratio FaDu/MeWo was 2.6 ± 0.9, *p* = 0.0194; n = 5, the one-sample t-test (µ = 1)). Moreover, we assessed the expression of *α_v_β_6_* integrin in 3D spheroids by immunofluorescent staining of cryosections (Figure 3b). Here, we used advanced stroma-rich 3D spheroid of HNSCC consisting of FaDu tumor cells and MeWo cancer-associated fibroblasts (CAFs), which were recently characterized in detail [30]. Using the liquid overlay technique, monoculture (F5) spheroids were generated from 5000 FaDu cells, while the co-culture (F5M5) spheroids consisted of 5000 FaDu cells and 5000 MeWo cells. As seen from the images, *α_v_β_6_* integrins are strongly expressed in FaDu cells in both monoculture (F5) and co-culture (F5M5) spheroids (Figure 3b, in red). In co-culture F5M5 spheroids, we observed the areas with lower fluorescence intensity, corresponding to MeWo clusters (Figure 3b, in green). MeWo cells were pre-stained with PKH67 membrane dye in order to distinguish CAFs in the co-culture spheroids.

#### 2.2.2. Uptake of QDs in 2D Monolayer Cells

First of all, we assessed the cytotoxicity of QDs on 2D monolayer FaDu and MeWo cells by MTT assay. The metabolic activity of cells was not affected after 24 h of incubation with 100 nM of QDs (*p* > 0.3, using the one-sample t-test with μ = 1). In fact, the viability of FaDu cells was 87 ± 15% and 91 ± 13% after incubation with QD-A20 and QD-SPP, respectively. In the case of MeWo cells, we observed 103 ± 7% (QD-A20) and 89 ± 23% (QD-SPP) of viable MeWo cells. It is worth noting that In-based QDs are considered as a less-toxic alternative to the first generation of NIR-emitting QDs.

**Figure 4:**
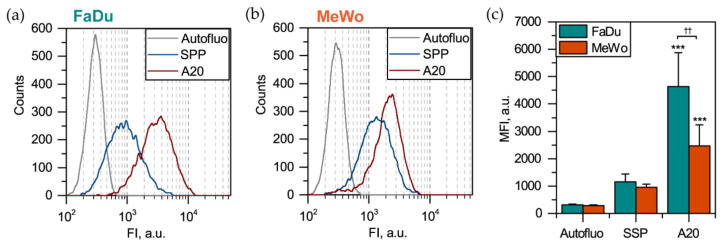
Uptake of QDs in 2D monolayer FaDu and MeWo cells. Typical cytometry distribution histograms of (**a**) FaDu and (**b**) MeWo monolayer cells 3 h post-incubation with 50 nM of QDs. (**c**) Mean fluorescence intensity (MFI) of FaDu (cyan bars) and MeWo (orange bars) cells 3 h post-incubation with QDs (n = 6–7; *** *p* < 0.001 compared to autofluorescence, using ANOVA; ^††^ *p* < 0.01, using two-sample t-test).

For uptake measurements, 2D monolayer cells were exposed to QD-A20 and QD-SPP for 3 h and analyzed by flow cytometry (Figure 4). As anticipated, the QD-A20 efficiently targeted both FaDu (Figure 4a) and MeWo (Figure 4b) cells due to the presence of *α_v_β_6_* integrins on their surface, compared to non-targeted QD-SPP, which tend to bind non-specifically to cell membranes (*p* < 0.001, using ANOVA) (Figure 4c). Moreover, the mean fluorescence intensity (MFI) of FaDu cells exposed to QD-A20 was significantly higher (*p* < 0.01, using a two-sample t-test) than that of MeWo cells. It is worth noting that all cells in 2D monolayer culture are equally available for QDs. Thus, the number of labeled cells tends to 100% (single-peak distribution histograms in panels A and B, Figure 4).

#### 2.2.3. Uptake of QDs in 3D Spheroids

**Figure 5:**
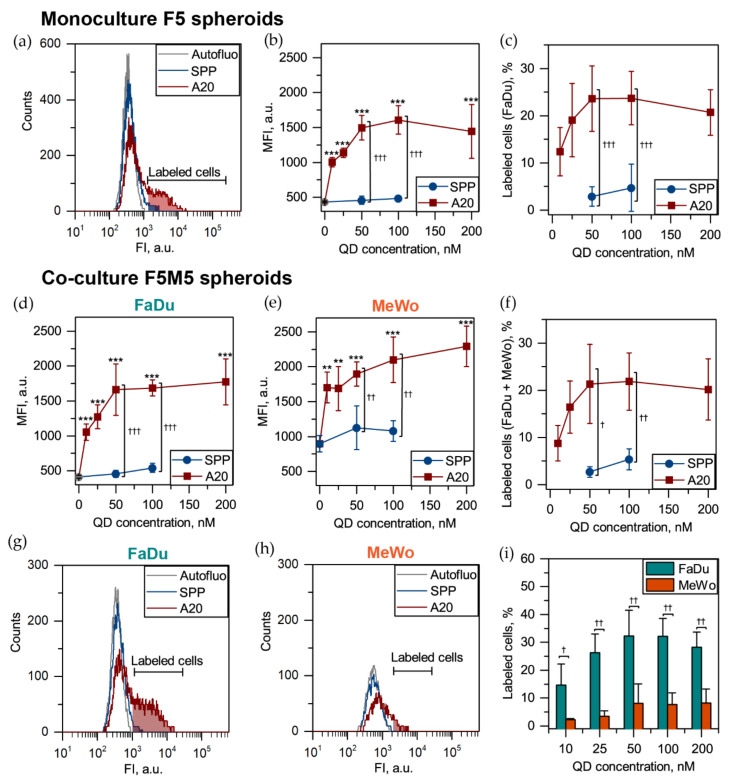
Uptake of QDs in 3D spheroids. (**a**) Flow cytometry histograms of cells from monoculture (F5) spheroids exposed for 3 h to 50 nM QD-SPP (blue) and A20-QDs (red). (**b**) The MFI and (**c**) the number of labeled FaDu cells in F5 spheroids exposed to various concentrations of QD-SPP (blue) and QD-A20 (red). MFI of (**d**) FaDu; (**e**) MeWo cells and (**f**) total number of labeled cells in F5M5 spheroids in the function of QD concentration. Typical flow cytometry histograms of (**g**) FaDu and (**h**) MeWo cells from F5M5 spheroids exposed for 3 h to 50 nM QD-SPP (blue) and A20-QDs (red). (**i**) The fraction of FaDu (cyan) and MeWo (orange) cells from F5M5 spheroids in the function of QD concentration. Data represent mean ± SD (n = 4–7; ** *p* < 0.01; *** *p* < 0.001 compared to autofluorescence, using ANOVA; ^†^ *p* < 0.05, ^††^ *p* < 0.01 and ^†††^ *p* < 0.001, using two-sample t-test).

There is growing evidence that 3D in vitro cell cultures better mimic the heterogeneity of in vivo tumors [31,32]. With this purpose, we tested the developed QDs in various concentrations in the F5 monoculture and F5M5 co-culture HNSCC spheroids. Compared to 2D monolayer cells, QD-A20 labeled only a limited fraction of cells in spheroids (Figure 5a). The detailed analysis demonstrated the saturation of the cellular uptake of QD-A20 in FaDu cells of F5 spheroids exposed up to 50 nM of QDs (Figure 5b). Moreover, a similar saturation effect was also observed for the percentage of labeled FaDu cells of 3D spheroids (Figure 5c). We also confirmed the dose-independent uptake of QD-A20 in co-culture FaDu/MeWo (F5M5) spheroids (Figure 5d,e). MeWo cells were priory stained with PKH67 membrane dye, thus displaying increased autofluorescence compared with 2D monolayer culture (Figure 5e). We showed that 50 nM, as well as 100 nM of QD-A20, labeled up to 25% of cells in spheroids, whereas QD-SPP—only up to 3 (50 nM) and 5% (100 nM). The thresholds of “labeled” cells were manually set to cover not more than 1% of cells in autofluorescnece (no drug) sample. The thresholds were calibrated separately for FaDu and MeWo cells in each independent experiment. Furthermore, QD-A20 preferably labeled FaDu cells in F5M5 spheroids for all QD concentrations (Figure 5g). At 50 nM, QD-A20 were detected in 32% of FaDu cells and in 8% of MeWo cells. As seen in Figure 5g,h, in co-culture spheroids QD-A20 labeled preferably FaDu, but not MeWo. Figure 5i displayed that FaDu cells better accumulate QD-A20 than MeWo cells irrespective of QD-A20 concentration. Of note, at 5-day post-seeding F5M5 spheroids consist of 65% of FaDu and 35% MeWo cells (Figure S4). Overall, the total fraction of QD-A20 labeled cells (100 nM) was similar in F5 and F5M5 spheroids (23.7 ± 5.7% vs. 21.8 ± 6.1%, *p* > 0.05; using the two-sample t-test). Importantly, no significant difference (*p* > 0.05; using ANOVA) was observed between autofluorescence and QD-SPP fluorescence for all types of spheroids (Figure 5a). Finally, to compare the specificity of QD-A20 to FaDu and MeWo cells in F5M5 spheroids, we subtracted the autofluorescence of FaDu and MeWo cells (Figure S5). Thus, we confirmed a higher specificity of QD-A20 to FaDu tumor cells than that to MeWo (CAFs) in 3D co-culture F5M5 spheroids (*p* < 0.05, using ANOVA).

#### 2.2.4. Penetration of QD-A20 in 3D Spheroids

**Figure 6:**
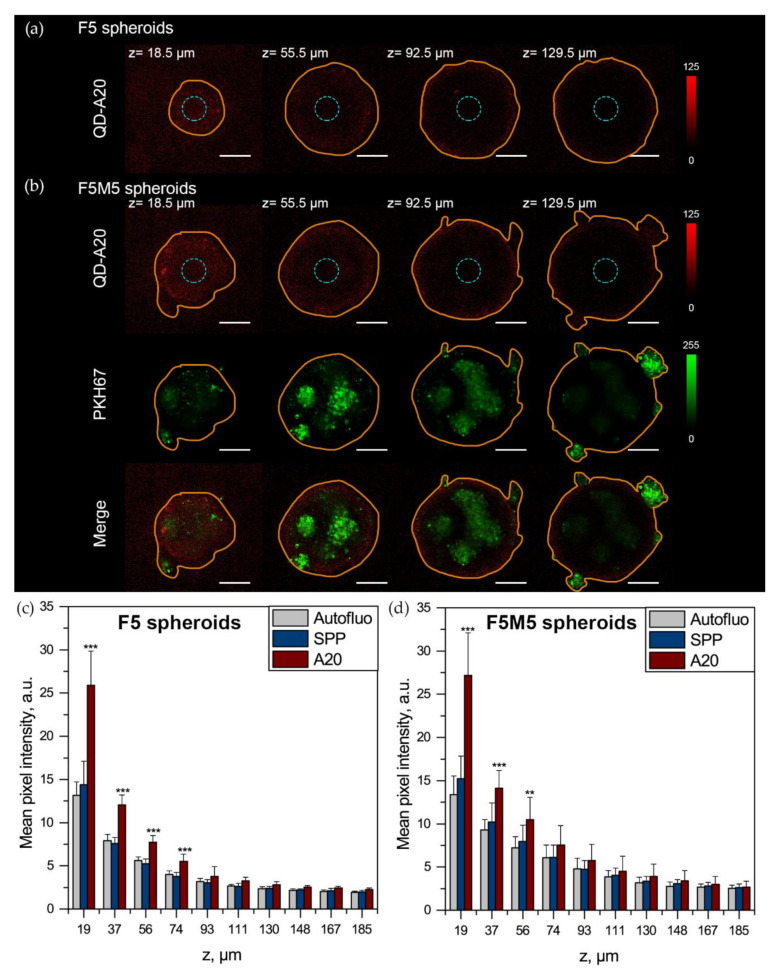
3D Fluorescence imaging of QD-loaded head and neck squamous cell carcinoma (HNSCC) spheroids. 3D confocal microscopy of QD-A20 (red color, λ_em_ = 730–800 nm) distribution in different optical sections of (**a**) F5 and (**b**) F5M5 spheroids, incubated for 3 h with 50 nM of QDs. MeWo cells were pre-stained with PKH67 membrane dye (green color, λ_em_ = 530–600 nm). The orange line displays the contour of spheroid. The mean pixel intensity of the central region (blue circles) of (**c**) F5 and (**d**) F5M5 spheroids 3 h post-incubation with 50 nM of QDs as a function of the depth (z) of optical section. Scale bar = 100 µm. Data represent mean ± SD (n = 3–7; ** *p* < 0.01; *** *p* < 0.001 compared to autofluorescence, using ANOVA).

The flow cytometry analysis of dissociated spheroid does not allow the cells near the center to be distinguished directly from the cells on the periphery of spheroids. Thus, the penetration of QDs in FaDu/MeWo spheroids was visualized by confocal laser scanning microscopy by excitation at 488 nm and registration at 730–800 nm. The Z-stacked confocal images from the spheroid surface to the center are shown in Figure 6a,b, providing better insight into the penetration of QDs in 3D spheroids. In our previous report, we demonstrated that five days post-seeding, the diameter of F5 and FM5 spheroids was 427 ± 18 μm and 478 ± 22 μm, respectively [30]. According to the fluorescence confocal images, after 3 h of incubation, QD-A20 were accumulated by cells on the periphery of both types of spheroids. Thus, to quantify the detection depth (λ_ex_ = 488 nm) of QDs in spheroids, we calculated the mean pixel intensity of the central part of each optical section (blue circle of 70 pixels in diameter) as a function of z-depth (Figure 6c,d). For F5 spheroids, the detection limit for QD-A20 was 74 µm from the periphery for 488 nm excitation wavelength (*p* < 0.05; using ANOVA) (Figure 6c). Of note, the observed signal for spheroids exposed to QD-SPP was similar to the autofluorescence of cells for all z-stack optical sections (*p* > 0.05; using ANOVA). In the case of F5M5 spheroids, we detected QD-A20 up to 56 µm from the periphery (*p* < 0.05; using ANOVA) (Figure 6d). Given the limited fraction of labeled cells detected by flow cytometry and peripherical QD distribution observed by fluorescence microscopy, it seems unlikely that cells in the center of spheroids accumulate a detectable amount of QDs.

#### 2.2.5. Time-Gated Fluorescence Imaging of 3D Spheroids in Tissue-Like Conditions

**Figure 7:**
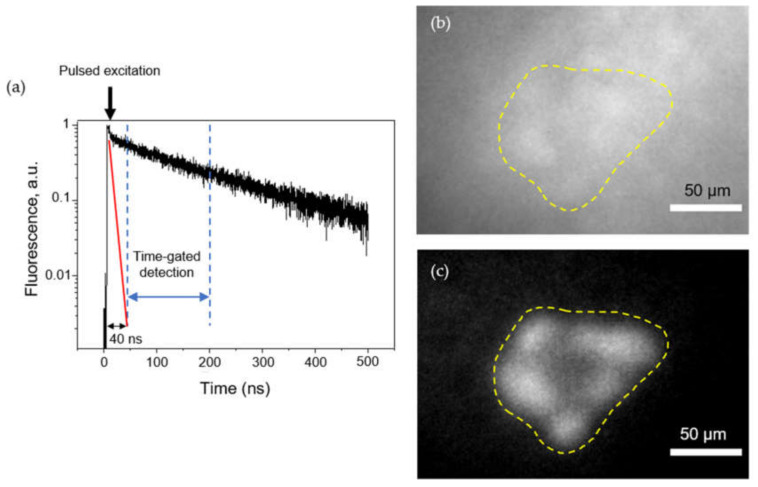
Time-gated imaging of QD-loaded co-culture HNSCC spheroids. (**a**) Autofluorescence (red) and fluorescence of QD-loaded spheroids (black) as a function of gate delay ∆t (gate duration: 160 ns). The first dotted line corresponds to the 40 ns delay selected for the following experiments. Typical fluorescence images at (**b**) delay = 0 and (**c**) 40 ns, showing QD-loaded F5M5 spheroids deposited on a slice of tissue (scale bar = 50 μm). The yellow dotted line displays the contour of F5M5 spheroid.

While in vitro fluorescence cell detection is very sensitive, the detection of very small tumor nodules in vivo is much more challenging due to the strong autofluorescence of tissues, providing a high non-uniform background. Long lifetime fluorescent QDs, coupled with time-gated imaging, afford a possibility for deep imaging. Introducing a controllable delay between the excitation pulse and the detection window (Figure 7a) enables efficient rejection of fast (≈ ns) autofluorescence photons and detection of slower (≈ 100–300 ns) QD fluorescence. As a proof of principle, we deposited F5M5 spheroids labeled with A20-QD onto mm-thick tissue slices to mimic tissue-like conditions. Figure 7b shows that in the absence of time gating, the autofluorescence background prevents the identification of the tumor spheroid labeled with a small concentration of QDs (50 nM). In contrast, when Δt = 40 ns delay is used between excitation and detection, the autofluorescence background is completely eliminated, revealing the presence of QD-labeled spheroid. For example, in the images shown in Figure 7, the signal-to-noise ratio (i.e., the ratio between signal—fluorescence intensity from the spheroid region after removal of average background—and noise—fluctuations of background autofluorescence) is 0.8 in the absence of time-gating, and around 20 with time-gating, thus improving sensitivity by a factor of 25 (Figure S6). Time-gated imaging requires a pulsed excitation source, in contrast to continuous sources used in other experiments. Our pulsed excitation source emits at 659 nm, but any other wavelength would work as well, as long as it falls within the QD extinction spectrum. Overall, we can conclude that selective labeling of spheroid by QDs in tissue-like conditions can make QDs a suitable tool for targeted intraoperative imaging of *α_v_β_6_* integrin-rich HNSCCs.

## 3. Discussion

In recent years, NIR-emitting QDs have emerged as a promising tool in analytical applications, especially for in vivo imaging and therapy [33,34,35]. Here, we report the development and validation of QD-A20 bioconjugates as nanoprobes for noninvasive NIR imaging of *α_v_β_6_* integrin-rich HNSCC. Experiments with 2D monolayer and 3D spheroid HNSCC cultures consisting of the tumor (FaDu) and CAF (MeWo) cells indicated that the QD-A20 nanoprobe is highly specific to *α_v_β_6_* integrin. Overall, QD-A20 demonstrated a higher selectivity for FaDu than that for MeWo cells. In fact, we also demonstrated a 2.5-fold overexpression of *α_v_β_6_* integrin in FaDu cancer cells compared to MeWo CAFs (Figure 3). The overexpression of *α_v_β_6_* in FaDu cells confirmed the previously published reports [36]. It is worth noting that A20FMDV2 peptide is highly specific only for *α_v_β_6_* but not for other RGD-recognizing integrins (e.g., *α_v_β_3_* and *α_v_β_5_*) [23]. Accordingly, we conclude that targeted QDs-A20 are selective to the HNSCC cells overexpressing *α_v_β_6_* integrin.

In clinical situations, tumor cells are organized as complex 3D tissue and associated with other cell types such as CAFs or macrophages. However, in the design of tumor-targeted nanoprobes, most in vitro studies focus on the labeling of tumor cells in 2D cultures. Moreover, there is increasing evidence that stroma could act as a physical barrier for nanoprobes restricting their penetration into the tumor stroma [37]. It is, therefore, critically important to characterize the behavior of tumor-targeted nanoprobes in the stroma-rich 3D model, in particular the extent to which they can selectively bind tumor vs. stromal cells and their capacity to penetrate in 3D tumor tissues. Thus, we tested QD-A20 in the advanced stroma-rich 3D model of HNSCC. This model recapitulates various levels of stroma expression in the HNSCC, contributing to the study of specific interactions of photoactive probes with stroma-rich HNSCC tissue. Due to the high binding specificity of QD-A20, the optimal concentration for NIR imaging with QD-A20 nanoprobe was quite low, equivalent to 50 nM of QDs. QD-A20 efficiently labeled about 25% of total cells in the outer layers of stroma-rich co-culture spheroids; most of them were FaDu cancer cells (89 ± 7% of total labeled cells). The saturation of QD-A20 uptake in cells is the obvious consequence of active targeting of a limited number of ligands—e.g., *α_v_β_6_* integrins, expressed on the surface of the cells [38]. Given the minimal toxicity of ZnCuInSe/ZnS core/shell, along with surface coating with SPP and low optimal concentration, we anticipated that QD-A20 could be compatible with clinical application.

Our results suggest the presence of diffusion limitations for the transport of QDs into the inner spheroid region. The obtained results confirmed literature reports on the limited (up to 100 µm) penetration of QDs into the 3D tumor spheroids [39,40,41]. For instance, Jarockyte and co-workers used mathematical modeling of QD penetration in spheroids and suggested that active transport plays a major role in QD penetration, whereas the diffusion of QDs through the ECM has no significant impact [39]. According to our data, the presence of ECM slightly decreased the detection depth of QD-A20 in spheroids acting as a physical barrier, as was assumed in the literature [37]. Nevertheless, the intravenous injection of QDs will allow selective staining of perivascular regions of the tumor. In fact, HNSCC are highly vascularized on the borders with normal tissues; thus, we may suppose an efficient application of developed QDs for delimitation of tumor borders. Despite the observed limited penetration of QD-A20 in spheroids, we could clearly detect the labeled tumor spheroids in tissue-like conditions using time-gated fluorescence microscopy due to the long lifetime of QD fluorescence. Such conditions do not remove nonspecific labeling from surrounding tissues. However, as shown in our manuscript, it significantly improves the sensitivity of detection vs. autofluorescence. While nonspecific QD signal in surrounding tissues would have to be estimated in vivo, we note that the high contrast obtained between FaDu (positive) and MeWo (negative) cell lines is encouraging for future in vivo targeting experiments.

## 4. Materials and Methods

### 4.1. Materials

N_3_-GGGSGGGSVPNLRGDLQVLAQKVART (N_3_-A20) peptide (>95% purity) was synthesized on the IBPS platform (Sorbonne University, Paris, France). Chemicals for the synthesis and functionalization of QDs were purchased from Sigma-Aldrich (Saint-Quentin Fallavier, France).

### 4.2. Synthesis of QDs

QDs were synthesized by solvothermal methods according to previously published protocols [11]. Briefly, Zn-Cu-In-Se cores were synthesized by the reaction of zinc acetate, copper chloride, indium chloride and selenouera in octadecene (ODE) at 260 °C, then a ZnS shell was grown by injection of zinc oleate and zinc diethyldithiocarbamate at 190 °C in ODE, as described previously [11]. To solubilize these QDs in water, 3-[3-methacrylamidopropyl-(dimethyl)-ammonio]propane-1-sulfonate (sulfobetaine, SPP), aminopropylmethacrylamide (APMA) and 4-vinylimidazole (VIM) monomers were used to synthesize p(SPP-b-VIM) and p((SPP-co-APMA)-b-VIM) block polymers by RAFT polymerization, as described earlier [21,28]. QDs were solubilized into water using mercaptopropionic acid, a short labile ligand, then exchanged in a second step with p(SPP-b-VIM) or p((SPP-co-APMA)-b-VIM) polymers. They were purified by ultracentrifugation in sucrose gradients and ultrafiltration [21]. Functionalization with the *α_v_β_6_*-targeting peptide was typically performed as follows: 3 nmol of QDs capped with p((SPP-co-APMA)-b-VIM) was re-suspended in 300 µL HEPES/NaCl buffer (10 mM, pH 7.5) and reacted with dibenzocyclooctyne-NHS ester (DBCO-NHS) (1.5 µmol; 5 g/L in DMSO) at room temperature for 2 h. QDs were purified from excess DBCO-NHS by ultrafiltration (3 rounds, 100 kDa cut-off, 14,000 *g*, 12 min). They were then mixed with 300 nmol of N_3_-A20 for 16 h at room temperature and purified by three rounds of ultrafiltration. The number of accessible NH_2_ and DBCO units per QD was evaluated by reacting the QDs at each step with Cy5-NHS and Cy5-N_3_ dyes, respectively, for 6 h at a 100:1 dye:QD ratio, purifying them with 3 rounds of ultrafiltration and measuring the dye:QD ratio by absorbance.

### 4.3. Cell Lines

The FaDu (human pharynx squamous cell carcinoma) cell line was purchased from ATCC (Cat. No: ATCC HTB-43). Cells were cultured in phenol red-free Roswell Park Memorial Institute 1640 medium (RPMI-1640, Invitrogen, Carlsbad, CA, USA), supplemented with 9% (*vol*/*vol*) heat-inactivated fetal bovine serum (FBS, Sigma-Aldrich, Saint-Quentin Fallavier, France), penicillin (10,000 IU) streptomycin (10,000 mg/mL) and 1% (*vol*/*vol*) 0.2 M glutamin (Invitrogen, Carlsbad, CA, USA). As CAFs, we used MeWo cells (ATCC HTB-65), granular fibroblasts derived from human melanoma [42], cultured in Minimal Essential Medium (MEM, Sigma-Aldrich, Saint-Quentin Fallavier, France), supplemented with 9% (*vol*/*vol*) of FBS and 1% (*vol*/*vol*) 0.1 M sodium pyruvate (Sigma-Aldrich, Saint-Quentin Fallavier, France). Monolayer cells were kept in a humidified incubator (5% CO_2_) at 37 °C. To ensure exponential growth, cells were reseeded every week.

### 4.4. Spheroids Formation

Spheroids were generated from FaDu cells using the liquid overlay technique, as described previously [30]. Briefly, to form monoculture (F5) spheroids, 200 µL of FaDu cells (2.5 × 10^4^ cells/mL) was added to each well of a 96-well plate previously coated with 1% agarose (*w/v* in water) and cultured at 37 °C, 5% CO_2_.

Co-culture (F5M5) spheroids were formed by seeding FaDu cells (100 µL at 5 × 10^4^ cells/mL) simultaneously with 100 µL of MeWo cells at 5 × 10^4^ cells/mL. To distinguish cells of different types in co-culture spheroid, MeWo cells were pre-stained with a membrane green fluorescent cell marker PKH67 (Sigma-Aldrich, Saint-Quentin Fallavier, France) according to the manufacturer’s recommendations, before seeding with FaDu cells.

At day 5 post-seeding, between 4 and 8 spheroids were used for each experimental condition. For immunohistochemistry analysis, spheroids were embedded into resin Shandon Cryomatrix (ThermoFisher, Waltham, MA, USA), frozen, cut and 10 µm thick sections were further analyzed by fluorescence microscopy. We used the cryosections with the diameter of the spheroid section about 400–450 μm.

### 4.5. Fluorescence Staining

For incubation of spheroids with QDs, 100 µL of the complete medium in each well with spheroids was carefully replaced with 150 µL of concentrated QD solution, prepared in FBS-free medium. Spheroids were kept in a humidified incubator (5% CO_2_) in the dark at 37 °C. At appropriate incubation time, spheroids were analyzed by fluorescence microscopy or flow cytometry after dissociation.

### 4.6. QDs Characterization

Fluorescence spectra and time-resolved fluorescence decays were acquired with a F-900 spectrometer (Edinburgh Instruments Ltd., Livingston, UK) equipped with a TCSPC card. The fitting function contains a background component. The best fit was obtained using the procedure included in the F-900 software. Absorbance spectra were registered using a Shimadzu UV-1800 absorption spectrometer. Dynamic light scattering (DLS) was performed on a Malvern CGS-3 goniometer system equipped with a HeNe laser (633 nm) and an ALV/LSE-5003 correlator (ALV-Laser Vertriebsgesellschaft m-b.H., Hessen, Germany). DLS measurements were performed using 3 runs of 30 s at 5 different angles.

### 4.7. Western Blot

Adherent cells were scrapped with ice-cold RIPA buffer 1X (Merck Millipore) supplemented with 20 µM anti-protease (Phenylmethanesulfonyl fluoride, PMSF, Sigma). Western blot analysis was performed with ninety microgram protein lysate from each cell line. Samples were loaded to 10% non-reducing SDS PAGE. After 90 min of blotting and saturation with 5% *w*/*v* solution of non-fat powdered milk in TBST for 1 h, the *β_6_* subunit of integrin *α_v_β_6_* was visualized. PVDF membrane was probed overnight at 4 °C with a rabbit anti-integrin *β_6_* specific antibody (clone 442.5C4; Abbexa; 1:500), or anti-tubulin antibody and followed by detection with appropriate HRP-conjugated secondary antibody (1:2000; Cell Signaling). Immune complexes were then detected by the chemiluminescence method and visualized with a gel imager (Azure C600, Azure Biosystems, Dublin, CA, USA). To compare the relative expression of *β_6_* in FaDu vs. MeWo cells, the band density was quantified using the ImageJ (NIH, USA) software and normalized to that of the tubulin.

### 4.8. Histology and Immunofluorescence Analysis

Cryosections were fixed with buffered 4% formaldehyde in PBS supplemented with sucrose 2% (*w*/*v*), rinsed two times with PBS, and permeabilized with 0.2% Triton X-100 for 5 min. Then, the sections were blocked with bovine serum albumin (3% *w*/*v*) in PBS for 1 h at room temperature. Slides were incubated with the primary antibody in the blocking solution overnight at 4 °C in a humidified chamber (dilution1:100). Antibody *α_v_β_6_* integrin was purchased by Biorbyt (Cambridge, UK). Samples were then extensively washed before indirect immunostaining with a secondary anti-rabbit antibody conjugated with Alexa 555 in PBS solution for 1 h at room temperature (dilution 1:200). After several washings, samples were mounted with a nuclear counterstaining solution with DAPI (Vectashield with DAPI, Vector laboratories, Burlingame, CA, USA) and then observed by fluorescence microscopy (AX-70 Provis, Olympus, Paris, France).

### 4.9. Cell Viability

The viability of cells exposed 24 h to 100 nM of QD-SPP and QD-A20 was estimated by MTT test. After washing with serum-free RPMI, 50 µL of MTT solution (2.5 mg/mL in PBS) was added to 100 µL of culture medium for 3 h at 37 °C. At the end of the incubation, the MTT was removed, and the crystals formed were dissolved with 50 µL of DMSO before absorbance measurements at 550 nm by a Multiskan spectrophotometer (Ascent, Thermo Fisher Scientific, Waltham, MA, USA).

### 4.10. Flow Cytometry

The cellular uptake of QDs in 2D monolayer cells and 3D spheroids was analyzed by flow cytometry. For this purpose, monolayer cells or spheroids were incubated with QDs for 3 h. The concentration of QDs was 50 nM for 2D monolayer studies. In the case of 3D spheroids, we incubated 10, 25, 50, 100, 200 nM of QD-A20 or 50, 100 nM of QD-SPP. Spheroids were further dissociated by their transfer into a 12-well plate, washed twice with PBS, and incubated with twice diluted trypsin-EDTA in PBS (Sigma-Aldrich, Saint-Quentin Fallavier, France). The plate was protected from light and placed on the rotatory shaker (60 rpm) for 20–25 min. To inhibit trypsinization, 3 mL of the complete culture medium was added to each sample. Finally, dissociated spheroids were re-suspended, centrifugated (1500 rpm, 5 min), and the pellet was re-suspended in the fresh serum-free culture medium. Cell suspensions were further subjected to flow cytometry analysis.

Flow cytometry analysis was performed using Accuri C6 Plus (BD, Franklin Lakes, NJ, USA), equipped with a laser emitting at 488 nm. The fluorescence of PKH67 was detected in the fluorescence channel FL1 with a 533 ± 30 nm filter under the excitation at 488 nm, while the detection of QD’s fluorescence was performed in an FL4 channel with 780 ± 60 nm filter (λ_ex_ = 488 nm). Data analysis was carried out using Accuri C6 Plus software (BD, Franklin Lakes, NJ, USA).

### 4.11. Laser Scanning Fluorescence Microscopy

Spheroids were incubated for 3 h in the dark at 37 °C with 50 μM of QDs, individually placed into Lab-Tek II chamber Slide (Roskilde, Denmark) and observed with a confocal laser-scanning microscope (Leica SP8 X AOBS LCSM, Leica microsystem, Wetzlar, Germany), equipped with an argon laser (λ_ex_ = 488 nm). Fluorescence images were recorded using an ×10, NA 0.4 dry objective. The QD fluorescence was collected at 730–800 nm, while the fluorescence of PKH67 at 520–570 nm. Image processing was performed with ImageJ (NIH, USA) software.

### 4.12. Time-Gated Fluorescence Microscopy

After 5 days of co-culturing, FaDu:MeWo spheroids were labeled with 50 nM QDs for 2 h, rinsed and deposited over a thick (>1 mm) slice of tissue to mimic in vivo imaging conditions. Time-gated imaging was performed using a 659 nm, 5 MHz, pulsed laser source, focused on the sample using a 20×, NA 0.7 water immersion objective. The fluorescence was collected through the same objective, separated by a dichroic and a long pass filter (715LP, Semrock). The image was acquired using a gated intensifier (Picostar HRI, LaVision) synchronized with the pulsed laser and a charged coupled device camera (Roper QuantEM 512C). Fluorescence images were acquired with a ca. 160 ns time gate with a tunable delay between the start of the time gate and the laser pulse. Full fluorescence images correspond to a situation where the laser pulse is included within the time gate and includes autofluorescence and QD signal. Time-gated fluorescence images were acquired with a ca. 40 ns time delay.

### 4.13. Statistics

The data from at least three independent experiments are presented as the mean ± standard deviation (SD). The one-sample t-test was used for statistical analysis of integrins expression in FaDu and MeWo cells with µ = 1 as H_0_. The statistical analysis of the two groups was performed with an unpaired, two-tailed t-test. Analysis of Variance (ANOVA) followed by Tukey’s multiple comparisons test was used for comparison of three or more groups. Data analysis was carried out with the Origin software (OriginLab Corporation, Northampton, MA, USA).

## 5. Conclusions

In this study, we report the development and validation of QD-A20 bioconjugates as nanoprobes for noninvasive NIR imaging of *α_v_β_6_* integrin-rich HNSCC. Experiments with 2D monolayer and 3D spheroid HNSCC cultures consisting of tumor (FaDu) and CAF (MeWo) cells indicated that the QD-A20 nanoprobe is highly specific to *α_v_β_6_* integrin. QDs-A20 were also tested in the advanced stroma-rich 3D model of HNSCC. Due to the high binding specificity of QD-A20, the optimal concentration for NIR imaging with a QD-A20 nanoprobe was quite low, equivalent to 50 nM of QD. Given the minimal-toxicity of ZnCuInSe/ZnS core/shell, surface coating with SPP and low optimal concentration, we anticipated that QD-A20 could be relatively nontoxic for clinical use. At the same time, QD-A20 efficiently labeled about 25% of total cells in the outer layers of stroma-rich co-culture spheroids; most of these were FaDu cancer cells (up to 90% of total labeled cells). Despite the observed limited penetration of QD-A20 in spheroids, we could clearly detect the labeled tumor spheroids in conditions close to in vivo using time-gated fluorescence microscopy, essentially due to the long lifetime of QD fluorescence.

It should be mentioned that further extensive studies are needed to confirm the ability of QD-A20 to image HNSCC in vivo, including pharmacokinetic properties and specificity of nanoprobe. Overall, we conclude that QD-A20 is a highly promising nanoprobe with an architecture suitably designed to preserve NIR emission and to accomplish a cellular targeting towards *α_v_β_6_* integrin receptors, thus paving the way for its potential application for NIR bioimaging and imaging-guided surgery.

## Supplementary Materials

The following are available online at https://www.mdpi.com/2072-6694/12/12/3727/s1, Figure S1: Colorimetric assay of the efficiency of the conjugation of the N_3_-A20 peptide to the accessible reactive moieties. Figure S2: Photoluminescence excitation spectrum from the NIR QDs (detection at 750 nm). Figure S3: Fluorescence decay of QD before and after functionalization with DBCO and A20 peptides. Figure S4: Flow cytometry histograms of FaDu and MeWo cells from co-culture (F5M5) spheroids exposed for 3 h to 50 nM A20-QDs. Figure S5: The adjusted MFI values (autofluorescnece subtracted) of FaDu and MeWo cells from co-culture (F5M5) spheroids exposed for 3 h to 50 nM of SPP-QDs and A20-QDs. Figure S6: Quantitative analysis of fluorescence images captured with and without time-dated detection.
