# Supplementary material for: NIR Imaging of the Integrin-Rich Head and Neck Squamous Cell Carcinoma Using Ternary Copper Indium Selenide/Zinc Sulfide-Based Quantum Dots
Source: Cancers (Basel). 2020 Dec 11;12(12):3727. doi: 10.3390/cancers12123727 (PMC7764319; doi:10.3390/cancers12123727)
Supplement: Supplementary file 1 [file cancers-12-03727-s001.pdf]

NIR imaging of the integrin-rich head and neck squamous cell carcinoma using ternary copper indium sulfide-based quantum dots

Ilya Yakavets [1], Aurelie Francois [1], Maelle Guiot [1], Nicolas Lequeux [2], Alexandra Fragola [2], Thomas Pons [2,*], Lina Bezdetnaya [1,*] and Frédéric Marchal [1,3]

[1] Université de Lorraine, CNRS UMR7039 CRAN, Institut de Cancérologie de Lorraine, 6 avenue de Bourgogne, 54519 Vandœuvre-lès-Nancy, France;

[2] Laboratoire de Physique et d'Étude des matériaux (LPEM, UMR 8213), ESPCI Paris, PSL University, CNRS, Sorbonne University, 10, rue Vauquelin, 75005 Paris, France;

[2] Surgical Department, Institut de Cancérologie de Lorraine, 6 avenue de Bourgogne, 54519 Vandoeuvre-lès-Nancy, France

Supporting Material

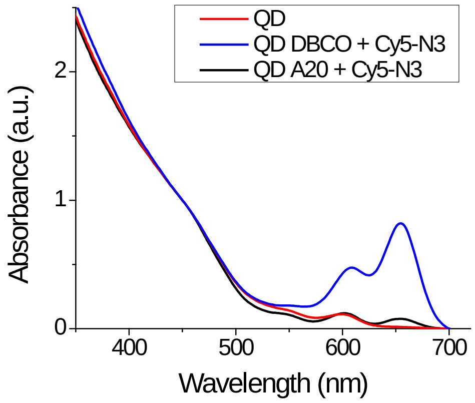

Figure S1. Colorimetric assay of the efficiency of the conjugation of the N_3-A20 peptide to the accessible reactive moieties. Red: Absorption spectra of visible emitting QDs before functionalization. The numbers of reactive DBCO groups available for reaction on QD-DBCO and remaining on QD-A20 after reaction with the N_3-A20 peptide were evaluated by reaction with excess N3-modified Cy5 dyes. After the reaction between the DBCO groups and the N3-dyes, the QDs were purified by several rounds of ultrafiltration. The comparison between the blue spectrum (QD-DBCO + N3-Cy5) and the black spectrum (QD-A20 + N3-Cy5) shows that the modification of DBCO groups with the peptide occurs with a >90 % efficiency.

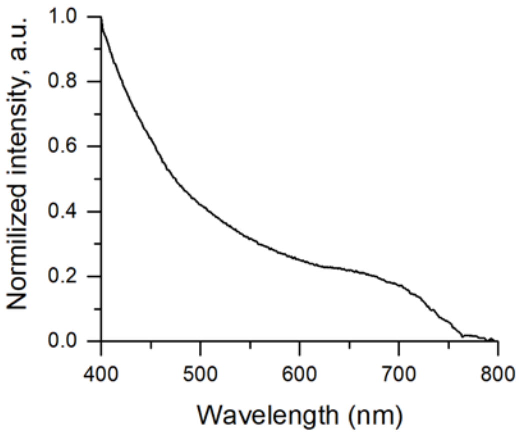

Figure S2. Photoluminescence excitation spectrum from the NIR QDs (detection at 750 nm).

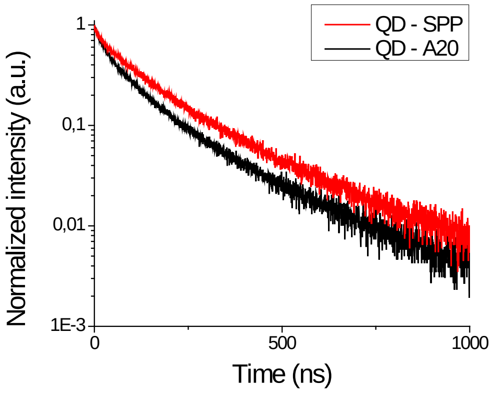

Figure S3. Fluorescence decay of QD before (red) and after (black) functionalization with DBCO and A20 peptides.

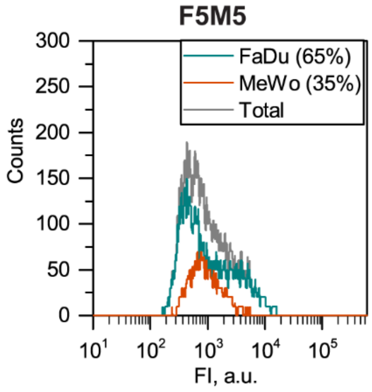

Figure S4. Flow cytometry histograms of FaDu (cyan) and MeWo (orange) cells from co-culture (F5M5) spheroids exposed for 3h to 50 nM A20-QDs.

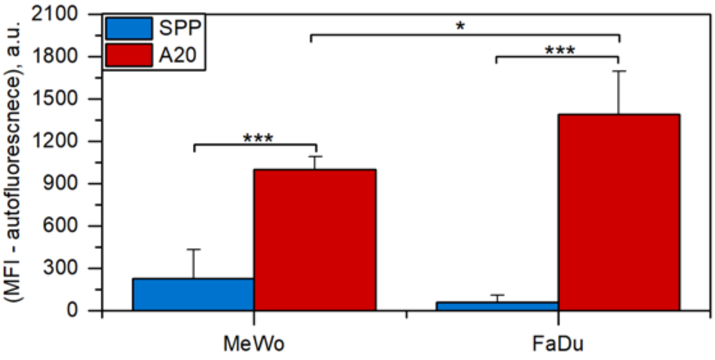

Figure S5. The adjusted MFI values (autofluorescence subtracted) of FaDu and MeWo cells from co-culture (F5M5) spheroids exposed for 3h to 50 nM of SPP-QDs (blue) and A20-QDs (red). Data represent mean ± SD [n = 4-6; *$p < 0.05$; ***$p < 0.001$, using ANOVA].

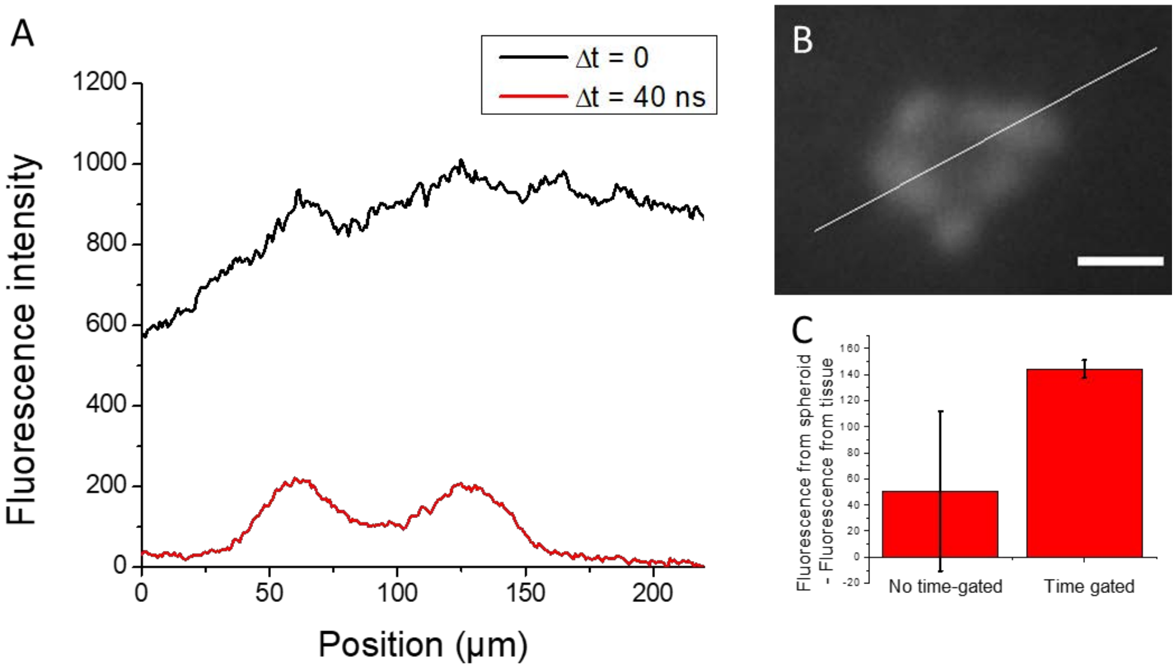

Figure S6. Quantitative analysis of fluorescence images captured with and without time-gated detection. (a) Fluorescence intensity along a line profile (shown in (b)) going through the spheroid without time-gated detection ($\Delta t = 0$, black) and with time-gated detection ($\Delta t = 40$ ns, red). (c) Comparison of the difference in average fluorescence intensity in the spheroid region and from the tissue outside of the spheroid without and with time-gated detection. The scale bar represents the standard deviation from the intensity pixel values from the tissue background.
